# Prokaryotic ancestry and gene fusion of a dual localized peroxiredoxin in malaria parasites

**DOI:** 10.15698/mic2015.01.182

**Published:** 2015-01-05

**Authors:** Carine F. Djuika, Jaime Huerta-Cepas, Jude M. Przyborski, Sophia Deil, Cecilia P. Sanchez, Tobias Doerks, Peer Bork, Michael Lanzer, Marcel Deponte

**Affiliations:** 1 Department of Parasitology, Ruprecht-Karls University, D-69120 Heidelberg, Germany.; 2 Structural and Computational Biology Unit, European Molecular Biology Laboratory (EMBL), D-69117 Heidelberg, Germany.; 3 Department of Parasitology, Philipps University, D-35043 Marburg, Germany.

**Keywords:** peroxiredoxin, molecular evolution, horizontal gene transfer, apicomplexa, malaria

## Abstract

Horizontal gene transfer has emerged as a crucial driving force for the evolution of eukaryotes. This also includes *Plasmodium falciparum* and related economically and clinically relevant apicomplexan parasites, whose rather small genomes have been shaped not only by natural selection in different host populations but also by horizontal gene transfer following endosymbiosis. However, there is rather little reliable data on horizontal gene transfer between animal hosts or bacteria and apicomplexan parasites. Here we show that apicomplexan homologues of peroxiredoxin 5 (Prx5) have a prokaryotic ancestry and therefore represent a special subclass of Prx5 isoforms in eukaryotes. Using two different immunobiochemical approaches, we found that the *P. falciparum* Prx5 homologue is dually localized to the parasite plastid and cytosol. This dual localization is reflected by a modular *Plasmodium*-specific gene architecture consisting of two exons. Despite the plastid localization, our phylogenetic analyses contradict an acquisition by secondary endosymbiosis and support a gene fusion event following a horizontal prokaryote-to-eukaryote gene transfer in early apicomplexans. The results provide unexpected insights into the evolution of apicomplexan parasites as well as the molecular evolution of peroxiredoxins, an important family of ubiquitous, usually highly concentrated thiol-dependent hydroperoxidases that exert functions as detoxifying enzymes, redox sensors and chaperones.

## INTRODUCTION

Parasitic lifestyles require dramatic changes of parasite genomes. An important option for such changes is the horizontal transfer of single or multiple genes across regular mating barriers 1,2,3. Accordingly, horizontal gene transfer following primary and secondary endosymbiosis has significantly altered the genomes of apicomplexan parasites including the human malaria parasite *Plasmodium falciparum* 4,5,6,7. In contrast to our knowledge on the acquisition of parasite genes from endosymbionts, only few examples for horizontal gene transfer from animal hosts or bacteria to apicomplexan parasites have been described 7,8,9,10,11,12. These examples support the importance of horizontal gene transfer for the evolution of apicomplexan parasites with regard to metabolic streamlining 8,9, the coordinated regulation of gene expression and the optimization of cytoadhesive domains on the parasite surface 11,12,13.

As apicomplexan parasites often occupy potentially harmful pro-oxidative ecological niches, such as macrophages or vertebrate erythrocytes, genetic events including horizontal gene transfer might also have shaped the parasites' peculiar redox metabolism. Peroxiredoxins are central players of the redox metabolism in pro- and eukaryotes. These usually highly concentrated enzymes detoxify hydroperoxides but can also exert functions as temporal redox sensors and chaperones 14,15,16,17,18. The genome of *P. falciparum* encodes five different peroxiredoxins 19, some of which have been shown to play a role in parasite development *in vivo *20,21. Furthermore, the parasite imports a highly abundant host peroxiredoxin for hydroperoxide removal 22. We recently characterized the so-called *P. falciparum* antioxidant protein (PfAOP) as a model enzyme for the Prx5 subfamily of peroxiredoxins *in vitro *23. PfAOP is rather unusual because of its ability to reduce hydrophobic hydroperoxides with glutathione and glutaredoxin as electron donors 23,24,25,26. So far, this enzyme has not been characterized *in vivo*. Here we show that PfAOP is dually localized to the parasite plastid and cytosol. The plastid localization is not the result of a gene acquisition by secondary endosymbiosis but presumably originates from a gene fusion event after a horizontal prokaryote-to-eukaryote gene transfer in a marine apicomplexan ancestor. These findings provide novel insights into the evolution of apicomplexan parasites and peroxiredoxins and might initiate the discovery of overseen horizontal gene transfer events.

## RESULTS

### PfAOP is dual localized to the apicoplast and cytosol 

**Figure 1:**
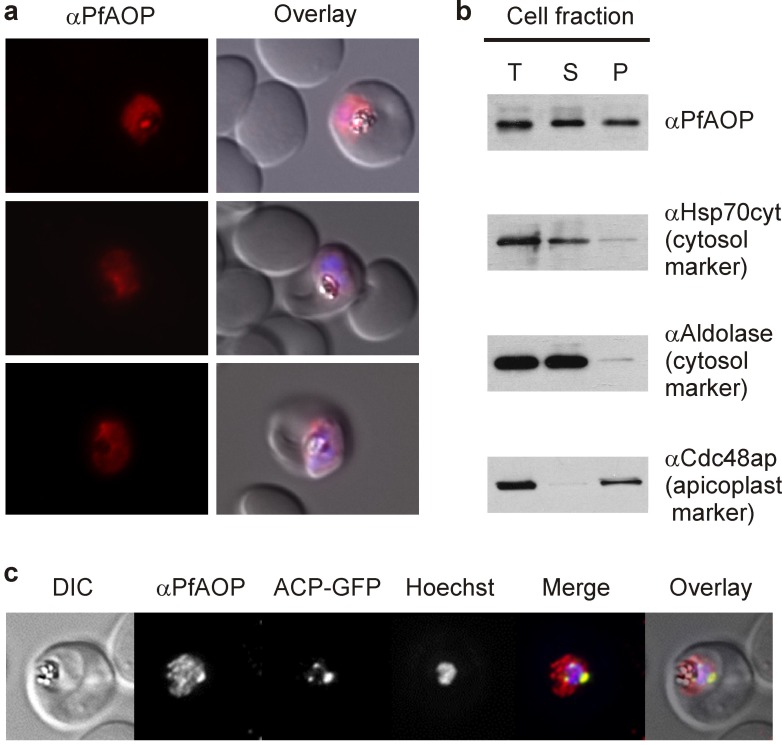
FIGURE 1: Dual localization of PfAOP. **(A)** Immunofluorescence localization of PfAOP in blood stage parasites. **(B)** Detection of PfAOP and marker proteins by western blotting after subcellular fractionation. The total parasite lysate (T), the supernatant (S) and the organellar pellet fraction (P) are shown from the left to the right side. PfAOP was detected at approximately 22 kDa (Fig S1). Apicoplast Cdc48, Hsp70 and aldolase were detected at approximately 130, 70 and 40 kDa, respectively. **(C) **Co-localization analysis of PfAOP and a chimera of acyl-carrier protein and GFP (ACP-GFP) as an apicoplast-localized marker protein.

PfAOP was previously predicted to localize to the apicoplast 25,26, and an episomally encoded fusion construct between GFP and the N-terminus of PfAOP without its Prx5 domain was indeed detected in this plastid organelle 27. When we analysed the subcellular localization of endogenous PfAOP by immunofluorescence microscopy, we observed punctate as well as network structures that are characteristic for the apicoplast 28. Much to our surprise, we furthermore detected a rather strong fluorescence in the parasite cytosol (Fig. 1A). Our purified peptide antibodies used for the localization experiments were specific for PfAOP (Fig. S1), and immunofluorescence microscopy with preimmune serum gave no signal under the same experimental conditions. We therefore hypothesised that PfAOP is dual localized. To further address this possibility, we generated *P. falciparum* cell extracts by saponin lysis and performed a subcellular fractionation. Subsequent western blot analyses revealed a dual localization of PfAOP in the cytosolic fraction as well as the organellar, apicoplast-containing fraction (Fig. 1B). Cytosolic Hsp70 and aldolase as well as Cdc48 in the apicoplast served as established marker proteins 29. The apparent size of PfAOP in both subcellular fractions was approximately 22 kDa and therefore slightly smaller than recombinant His-tagged PfAOP^∆59^ which runs at approximately 25 kDa 23 (Fig. 1B and Fig. S1). Unprocessed full length PfAOP with a calculated molecular mass of 28.1 kDa was not observed (Fig. S1). In addition, the punctate structures in Fig. 1A were confirmed to represent the apicoplast, as revealed by co-localization experiments with transgenic parasites that expressed a GFP-tagged apicoplast marker protein (Fig. 1C). In summary, PfAOP is not exclusively an apicoplast protein but is actually dual localized to the apicoplast and the parasite cytosol.

### A modular gene architecture reflects the dual localization 

**Figure 2:**
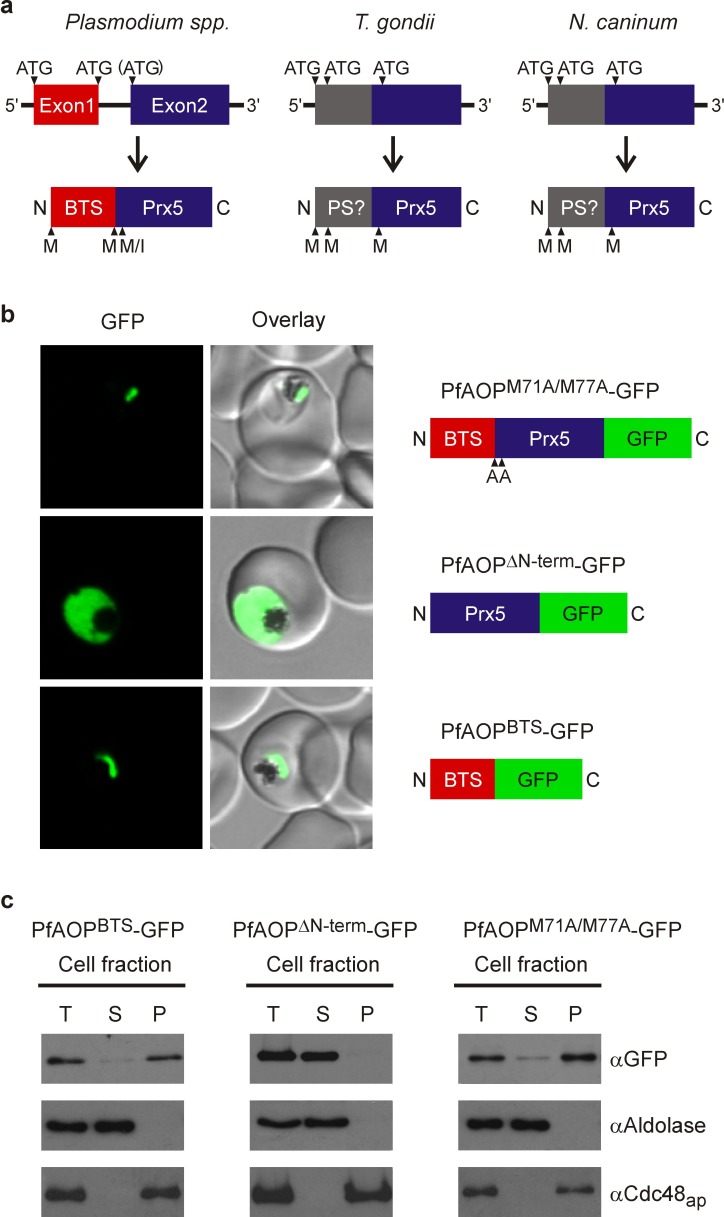
FIGURE 2: The modular gene architecture reflects the dual localization of PfAOP. **(A)** Schematic summary of gene and protein sequence comparisons between Prx5 isoforms from apicomplexan parasites. The targeting sequence of PfAOP and predicted pre-sequences (PS?) are indicated. The scheme is based on data bank entries TGGT1_038055, NCLIV_014020 and the entries given in Fig. S4. **(B)** Confocal live cell imaging of blood stage parasites expressing the indicated PfAOP-GFP chimera. **(C) **Subcellular fractionation and western blot analyses of the GFP-expressing strains from panel b. PfAOP^∆N-term^-GFP was detected at approximately 49 kDa in accordance with the calculated molecular mass. As expected for successful BTS-processing upon apicoplast import, the calculated/detected molecular masses for PfAOP^BTS^-GFP and PfAOP^M71A/M77A^-GFP were 35/25 and 55/49 kDa, respectively.

The gene architecture of PfAOP is conserved among *Plasmodium* species: Exon1 encodes the bipartite topogenic signal (BTS) consisting of an ER-type signal followed by a plant-like transit peptide required for targeting to the apicoplast 28. Please note that the BTS is usually processed upon protein import 28 in accordance with the observed size of mature PfAOP in Fig. 1B and Fig. S1. Exon2 encodes the Prx5 domain (Fig. 2A and Fig. S2). The BTS is necessary and sufficient to target PfAOP to the apicoplast, as revealed by fluorescence microscopy and subcellular fractionation assays with transgenic parasites expressing different GFP-tagged PfAOP constructs. Removal of the N-terminus in PfAOP^∆N-term^-GFP abrogated the apicoplast targeting, whereas constructs with mutated methionine residues at the start of the Prx5 domain (PfAOP^M71A/M77A^-GFP) or without the C-terminal Prx5 domain (PfAOP^BTS^-GFP) were found exclusively in the apicoplast (Fig. 2B,C and Fig. S3). To analyse the evolutionary conservation of the modular architecture of PfAOP and its *Plasmodium* homologues, we searched the genomes of other apicomplexan parasites for similar genes. We were able to identify Prx5 homologues in *Toxoplasma gondii* and *Neospora caninum* but not in *Cryptosporidium, Eimeria*,* Babesia* and* Theileria*. The Prx5 homologues from *Toxoplasma* and *Neospora* are encoded by a single exon (Fig. 2A) and have significantly altered N-termini (Fig. S4).

Furthermore, in contrast to their BTS-containing *Plasmodium* homologues, the *Toxoplasma* and *Neospora* proteins are predicted to localize to the mitochondrial matrix or the endoplasmic reticulum (using a variety of prediction programmes as listed in the methods section). In summary, Prx5 homologues are found in several apicomplexan parasites but the modular gene architecture of PfAOP with its separate BTS-encoding exon1 appears to be specific for *Plasmodium* species.

### Prokaryotic ancestry and gene fusion of PfAOP

**Figure 3:**
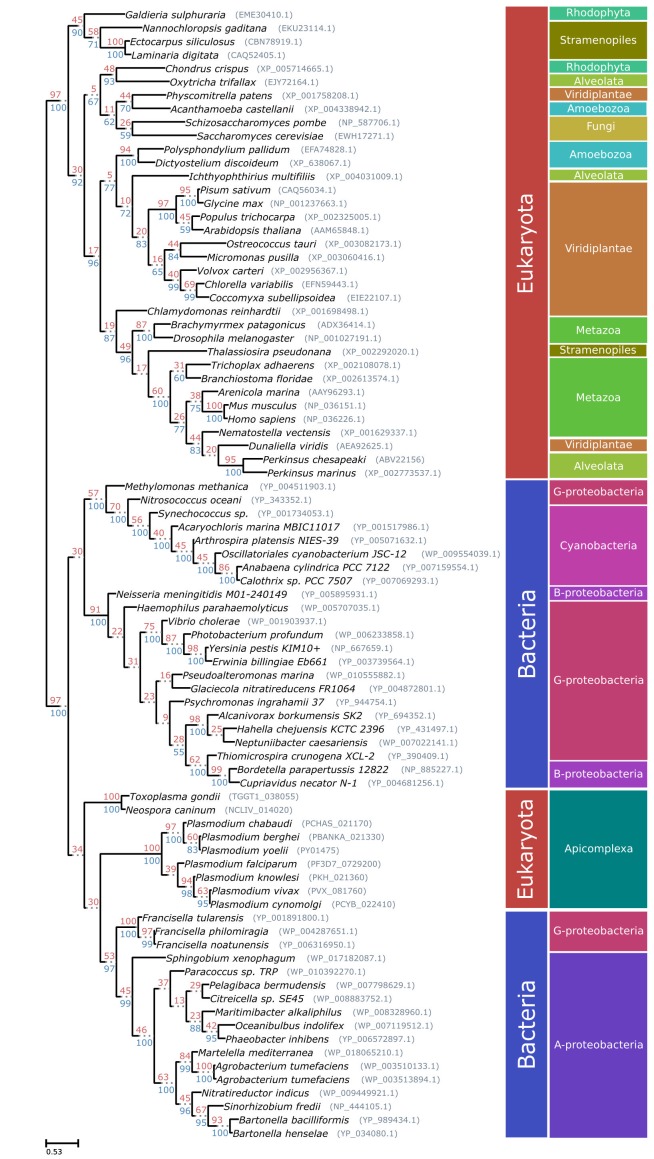
FIGURE 3: Phylogenetic tree of the Prx5 gene family. Maximum likelihood based phylogeny of 84 homologous Prx5 protein sequences. Bootstrap values are indicated in red on top of the branches. Posterior probability values, based on the Bayesian phylogenetic analysis are shown in blue for the branches shared with the maximum likelihood tree. Plasmodium sequences refer to the second exon of the PfAOP gene.

The apicoplast of apicomplexan parasites is a chloroplast-like organelle that was most likely acquired by secondary endosymbiosis after engulfment of a single-celled red alga by an auxotrophic protist 30,31. As a result, numerous genes of apicomplexan parasites, such as ApiAP2 transcription factors, the histone modifier Ashr3 and fatty acid synthases, probably have an algal origin 4,7,12,13. Since PfAOP carries a BTS, we wanted to analyse its phylogenetic origin and potential acquisition by secondary endosymbiosis. Although PfAOP and plant Prx5 isoforms share several properties on the protein level 23,24,32, PfAOP and its apicomplexan homologues did not cluster together with algal or plant homologues in phylogenetic sequence analyses (Fig. 3). In contrast, highest similarities were found between apicomplexan and bacterial Prx5 homologues. Even though the bootstrap values in the resulting phylogenetic tree were low, none of the bootstrapped trees recovered the apicomplexan clade as a monophyletic branch with any algae or alveolate species. We further discarded such hypotheses by testing 96 alternative tree topologies with different constrained positions for the monophyletic apicomplexan clade. The results were consistent regardless of the alignment trimming strategy or phylogenetic inference methodology used (i.e. maximum likelihood or Bayesian inference).

Altogether, even though the phylogenetic signal was not sufficiently strong to elucidate a clear ancestor for the PfAOP gene, our tests revealed a phylogenetic incongruence that excludes a horizontal gene transfer from an algal nucleus and that is in agreement with a direct prokaryotic ancestry of PfAOP and its apicomplexan homologues. Hence, the apicoplast localization of PfAOP is not a consequence of secondary endosymbiosis, but is presumably derived from the secondary addition of exon1.

Please note that we were unable to identify similar bacterial-like Prx5 homologues in dinoflagellates or other alveolates by BLAST searches. This excludes a mitochondrial ancestry of PfAOP and suggests a horizontal prokaryote-to-eukaryote gene transfer after the divergence of the apicomplexan and dinoflagellate lineages. (The few Prx5 homologues that were identified in other alveolates, including two *Perkinsus* species and the ciliates *Oxytricha trifallax* and *Ichthyophthirius multifiliis*, are in the exclusively eukaryotic branch of Fig. 3, which might point to a horizontal gene transfer resulting from secondary endosymbiosis for these selected genes). In summary, our data support a prokaryotic ancestry and contradict a plastid or mitochondrial origin of apicomplexan Prx5 homologues.

## DISCUSSION

When exactly might the prokaryote-to-eukaryote gene transfer of a Prx5 homologue have occurred? Among apicomplexan parasites, the blood parasites *Babesia* and* Theileria* are closely related to *Plasmodium* and are altogether classified as aconoidasida, whereas *Cryptosporidium* and the coccidia* Toxoplasma*, *Neospora* and *Eimeria *are conoidasida 6. The presence of similar Prx5 homologues in aconoidasida and conoidasida (Fig. 2A and Fig. 3) suggests that the gene was acquired before both parasite lines have diverged - which presumably occurred before the Cambrian radiation 33 - because a single gene acquisition followed by the loss of the bacterial-like Prx5 homologue in some apicomplexans is much more likely than independent gene acquisitions in *Plasmodium*, *Toxoplasma* and *Neospora*. A plausible evolutionary scenario is therefore a horizontal gene transfer between a marine bacterium and an ancestor of apicomplexans. Such an ancestor might have shared significant similarities with the closely related photosynthetic coral symbionts *Chromera velia* or *Vitrella brassicaformis *30,34, and it will be interesting to analyse whether these organisms possess Prx5 homologues of PfAOP. Corals would actually provide an excellent environment for a prokaryote-to-eukaryote gene transfer because they act as common hosts for bacterial and eukaryotic symbionts. Regarding the bacterial source of the apicomplexan Prx5 homologues, Fig. 3 may suggest an alphaproteobacterium, however, based on the bootstrap values, we cannot exclude another bacterial origin. After horizontal gene transfer, the BTS of the *Plasmodium *homologues was presumably acquired by the secondary addition of exon1. Such an evolutionary scenario is in agreement with a previous report on exon shuffling as a likely source for apicoplast targeting in *Plasmodium *35.

As far as the redox metabolism of *P. falciparum* is concerned, an important implication of the dual localization of PfAOP is that the reducing agents glutathione and cytosolic glutaredoxin are able to interact with PfAOP under physiological conditions *in vivo* in accordance with our previous mechanistic studies *in vitro *23. Furthermore, because of its dual localization, PfAOP could be a candidate not only for temporal redox sensing, as described previously for other peroxiredoxins 18, but for *spatio*temporal redox sensing in different subcellular compartments during the parasite's sophisticated life cycle.

In conclusion, our studies reveal a dual localization of the Prx5 homologue PfAOP in the parasite apicoplast and cytosol. The dual localization is reflected by a modular gene architecture, which appears to be conserved in *Plasmodium* but not in other apicomplexan parasites. PfAOP was not acquired by secondary endosymbiosis but presumably results from a gene fusion event after a horizontal prokaryote-to-eukaryote gene transfer in a marine apicomplexan ancestor. Hence, PfAOP and related proteins from *Toxoplasma* and *Neospora* form a novel subclass of Prx5 homologous with prokaryotic ancestry. In addition, PfAOP reflects an intriguing example for the addition of a gene by horizontal prokaryote-to-eukaryote gene transfer in an otherwise highly economised parasite genome. The physiological relevance and the advantage of targeting PfAOP to the apicoplast remain to be studied. Another aspect noteworthy is that some intracellular alphaproteobacterial or gammaproteobacterial pathogens, such as *Bartonella* or *Francisella* species, do not only possess Prx5 homologues that are extremely similar to the

apicomplexan homologues, but also share vertebrate host cells and arthropod vectors with apicomplexan parasites. It might therefore be promising to analyse potentially analogous Prx5 functions in these pathogens as well as putative pathogen-to-pathogen gene transfers upon co-infection.

## MATERIALS AND METHODS

### Cloning of PfAOP-GFP constructs

The full-length *pfaop *gene was PCR amplified from a cDNA library of *P. falciparum* strain 3D7 and cloned into the vector pDrive (Qiagen) using the restriction site-containing primers fl_PfAOP/pARL/s (5'-GCGC*CCATGG*TATAAATGAGAATGAGAAG AACAATAC-3') and PfAOP/pARL/as (5'-GCGC*CCTAGG*ACCATTA CCTAACTGATTATTTTTTAAAAACTCTTTTAC-3'). The truncation constructs *pfaop*^∆^*^Nterm ^*and *pfaop^BTS^*were amplified from full-length *pfaop* using the alternative primers sh_PfAOP/pARL/s (5'-GCGC*CCATGG*CAAAAGAAAATGATCTTATTCCTAACG-3') and BTS_PfAOP/pARL/as (5'-GCGC*CCTAGG*ACCATTAGGAATAAGAT CATTTTCTTTTATATC-3'), respectively. Potential alternative start codons in full-length *pfaop* were replaced by site-directed mutagenesis using the primers PfAOP-M71A/M77A/s (5'-CGTGAAAGTT*GCA*ATTGACGTAAGAAAT*GCA*AACAACAT ATC-3') and PfAOP-M71A/M77A/as (5'-GATATGTTGTT*TGC*ATTTCTT ACGTCAAT*TGC*AACTTTCACG-3'). Next, an *Nco*I restriction site in the GFP-coding sequence of *pfgloI-gfp*/pARL 36 was removed by site-directed mutagenesis using the primers GFP∆NcoI/s (5'-GAAAACTACCTGTTCC*T*TGGCCAACACTTGTCAC-3') and GFP∆NcoI/as (5'-GTGACAAGTGTTGGCCA*A*GGAACAGG TAGTTTTCC-3'). The gene *pfgloI* was subsequently excised using *Avr*II and *Nco*I, and full length and truncated *pfaop* was cloned into the vector pARL after digestion with the same restriction enzymes. Correct sequences of all *pfaop-gfp*/pARL constructs were confirmed by commercial sequencing (MWG Eurofins).

### *P. falciparum* culture and transfection

*P. falciparum* strain 3D7 was cultured at 37°C according to standard protocols 37 using fresh A+ human red blood cells at 3% hematocrit in RPMI 1640 medium that was supplemented with 0.45% (w/v) Albumax II, 200 µM hypoxanthine and 8.9 µg/µl gentamicin. Parasite cultures were maintained at 80% humidity, 5% CO_2_, 5% O_2_ and 90% N_2_. When necessary, parasites were synchronized using the sorbitol method 38. Transfections were performed as previously described 39 using 100 µg of plasmid DNA per construct. Parasites were subsequently selected with 5 nM WR99210. Transfectants were detected in blood smears between two and four weeks post transfection.

### Generation and purification of antibodies

Two rabbit peptide antibodies against residues 79-92 and 208-226 of PfAOP were generated (Pineda Antibody Service) and subsequently purified from sera by affinity chromatography using the peptides NH_2_-CNISDTDGSPNDFTS-CONH_2_ (αPfAOP-2) and NH_2_-CMFQEKDKQHNIQTDPYDIS-CONH_2_ (αPfAOP-5) according to an established protocol 40. The purity of the eluate fractions was verified by SDS-PAGE and the specificity of the peptide antibodies was assessed by immunoblotting against lysates of both uninfected and infected red blood cells. Preimmune sera served as controls (Fig. S1).

### Live cell imaging

Live parasites were washed twice with Ringer’s solution (122.5 mM NaCl, 5.4 mM KCl, 1.2 mM CaCl_2_, 0.8 mM MgCl_2_, 11 mM D-glucose, 10 mM HEPES, 1 mM NaH_2_PO_4_, pH 7.4) and visualized using a LSM510 confocal laser scanning microscope (Zeiss). GFP fluorescence was detected using an argon laser with 3% laser transmission, 40% output, 505-530 nm band pass filter and excitation at 488 nm. All images were processed with the software ImageJ 41 and are representative of at least 20 individual observations for each transgenic parasite line. A Gaussian filter of radius 1.0 was applied on all images. No gamma correction was performed.

### Immunofluorescence analyses

Indirect immunofluorescence microscopy was carried out as previously described 29 using αPfAOP-2 (1:50) and anti-rabbit Cy3 (1:2000) on either wild-type 3D7 or transgenic ACP-GFP 42 parasites. Preimmune serum at the same dilution gave no signal. Cells were counterstained with Hoechst 33258 (50 ng/ml) to visualise the nucleus. Fixed parasites were imaged on an epifluorescence Zeiss Cell Observer system, z-stack images were subjected to deconvolution (Zeiss Axiovision), maximally projected, converted to 8-bit TIFF format, pseudocoloured and overlayed (ImageJ, version 1.48). All images presented are representative of at least 20 individual observations.

### Parasite purification and subcellular fractionation

The subcellular fractionation of *P. falciparum* infected red blood cells was performed as previously described with slight modifications 43. Briefly, parasites at the trophozoite stage were purified by magnetic cell separation 44 on a VarioMACS column (Miltenyi Biotec) and released from red blood cells using 0.1% saponin in PBS (1.84 mM KH_2_PO_4_, 10 mM Na_2_HPO_4_, 137 mM NaCl, 2.7 mM KCl, pH 7.4). Freed parasites were re-suspended in PBS that was supplemented with protease inhibitor cocktail (Roche). Parasites were then lysed by five freeze-thaw cycles. The obtained homogenate, herein referred to as the total parasite lysate, was centrifuged (16,000 *g*, 60 min, 4°C) to separate the soluble supernatant fraction (S), which represents the cytosolic fraction, from the organelle-enriched pellet fraction (P). After removal of the supernatant, the pellet was washed twice with PBS. The three fractions were then mixed with Laemmli buffer supplemented with 30% 2-mercaptoethanol, boiled for 5 min at 95°C and analysed by SDS-PAGE and western blotting. The following primary antibodies were used at the indicated concentrations: αPfHsp70 (1:1000), αPfCdc48 (1:1000), αPfAldolase (1:5000), αGFP (1:2000), αPfAOP-2 (1:100) and αPfAOP-5 (1:200). Proteins were detected by chemiluminescence using goat anti-mouse HRP-conjugated secondary antibody (1:6000, Bio-Rad) for PfHsp70 and goat anti-rabbit HRP-conjugated secondary antibody (1:10000, Bio-Rad) for all other antibodies.

### Phylogenetic analyses and bioinformatics

All phylogenetic analyses were carried out using 84 homologous manually selected Prx5 amino acid sequences, covering 44 eukaryotes and 40 bacterial species. The first exon of the *Plasmodium* sequences was removed prior to all analyses. Multiple sequence alignment was produced using MAFFT v7 45 with the LINSi algorithm and a gap-opening penalty of 0.1. Evolutionary model selection was performed prior to phylogenetic inference using ProtTest v3 46 with default parameters, obtaining WAG as the best fitting model. Tree inference was carried out using both maximum likelihood (RAxML v8 47, rapid hill climbing algorithm, gamma distribution, WAG model, 100 runs and 100 bootstrap replicates) and Bayesian approaches (MrBayes 48, 4 chains, fixed WAG model, gamma distribution, 4 rates categories, 5,000,000 generations). A trimmed version of the alignment, generated with TrimAl v1.3 49 and gap threshold of 40%, was also evaluated, obtaining no significant differences in the topology and bootstrap supports. The relative position of the apicomplexan proteins was manually inspected for each of the 100 bootstrapped trees, obtaining 96 topologies in which the apicomplexan proteins were still grouped within the bacteria clade, and 4 topologies in which *Plasmodium* and coccidian species were split, the first being grouped as part of the eukaryotic clade and the former within bacteria. Similarly, all the sub-optimal topologies obtained from 100 RAxML runs were evaluated separately, obtaining no trees in which the apicomplexan proteins were recovered within the eukaryotic clade. Tree hypothesis testing was performed using CONSEL 50 on 96 alternative topologies covering all possible phylogenetic positions of the apicomplexan clade as a monophyletic group with any of the alveolate or algae species considered in the tree (http://github.com/jhcepas/prx5_supplementary_data . For this, 96 constrained topologies were manually generated and subsequently optimized using RAxML with the same parameters described above. All alternative phylogenies were evaluated with CONSEL. The 96 evaluated alternative topologies were rejected with P-values < 0.01 (73), < 0.05 (12) and < 0.1 (11) using the Approximately Unbiased (AU) test 51. Tree visualization, the generation of constrained tree topologies and the inspection of bootstrapped and sub-optimal tree topologies were performed using ad-hoc scripts based on the ETE toolkit 52. The complete list of topologies evaluated, source data, software and scripts are available as supplementary data at (http://github.com/jhcepas/prx5_supplementary_data . The subcellular localization of PfAOP and homologues from other alveolates were predicted using a variety of available bioinformatics tools including PATS 53, Predotar 54, PlasMit 55 and Wolf and iPSORT 56.

## SUPPLEMENTAL MATERIAL

Click here for supplemental data file.

All supplemental data for this article are also available online at http://microbialcell.com/researcharticles/prokaryotic-ancestry-and-gene-fusion-of-a-dual-localized-peroxiredoxin-in-malaria-parasites/.
