# Supplementary material for: Prokaryotic ancestry and gene fusion of a dual localized peroxiredoxin in malaria parasites
Source: Microb Cell. 2015 Jan 5;2(1):5–13. doi: 10.15698/mic2015.01.182 (PMC5361646; doi:10.15698/mic2015.01.182)
Supplement: Supplementary file 1 [file mic-02-005-s01.pdf]

Supplemental Data

Prokaryotic ancestry and gene fusion of a dual localized peroxiredoxin in malaria parasites

Carine F. Djuika, Jaime Huerta-Cepas, Jude M. Przyborski, Sophia Deil, Cecilia P. Sanchez, Tobias Doerks, Peer Bork, Michael Lanzer, and Marcel Deponte*

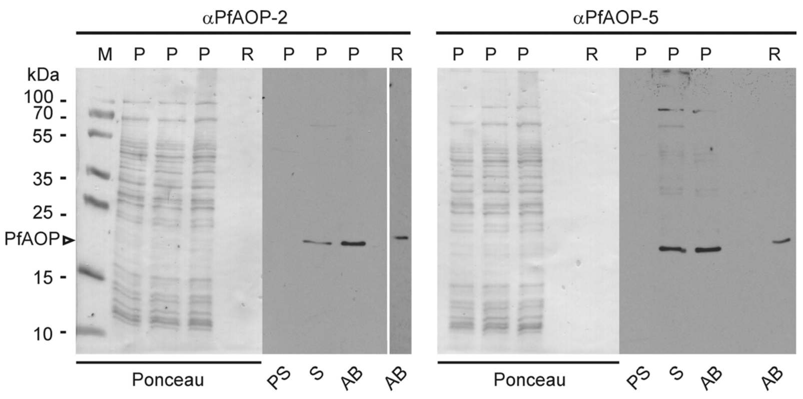

FIGURE S1: Generation, purification and analysis of rabbit peptide antibodies αPfAOP-2 and αPfAOP-5. A molecular weight marker (M), a *P. falciparum* extract of 10^7 cells (P), and 1 ng of recombinant His-tagged *Pf*AOP$^{\Delta 59}$ (R) were separated by reducing 15 % SDS-polyacrylamide gel electrophoresis and analysed by western blotting. The Ponceau-stained membrane on the left side served as a loading control. Results after decoration with either preimmune serum (PS), serum (S), or affinity-purified antibodies (Ab) are shown on the right side. False positive bands were absent at the chosen film exposure times, which yielded strong specific signals for both purified antibodies.

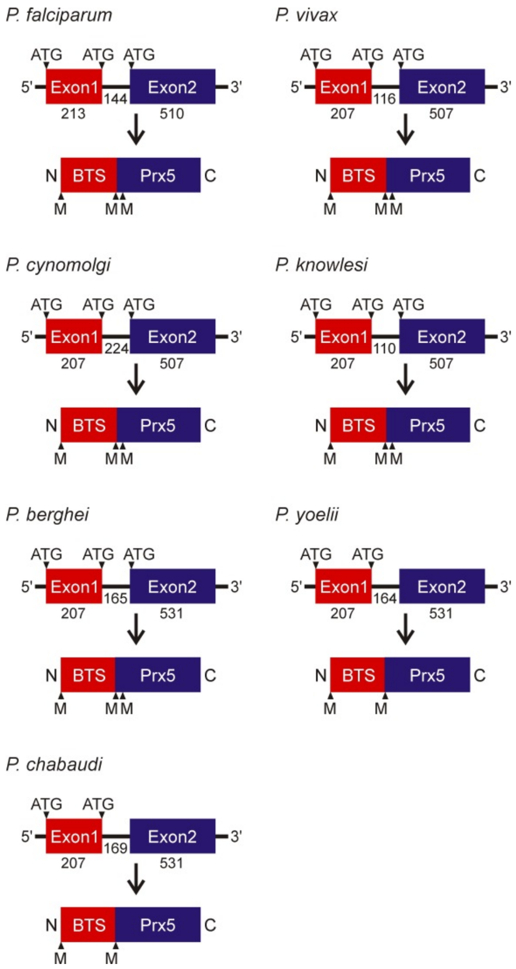

FIGURE S2: The modular gene architecture of PfAOP is conserved in malaria parasites. The following PlasmoDB entries were used for the generation of the schematic summary: *P. falciparum* PF3D7_0729200, *P. vivax* PVX_081760, *P. cynomolgi* PCYB_022410, *P. knowlesi* PKH_021360, *P. berghei* PBANKA_021330, *P. yoelii yoelii* PY01475, *P. chabaudi chabaudi* PCHAS_021170. Lengths of exon1, the intron and exon2 are indicated in base pairs.

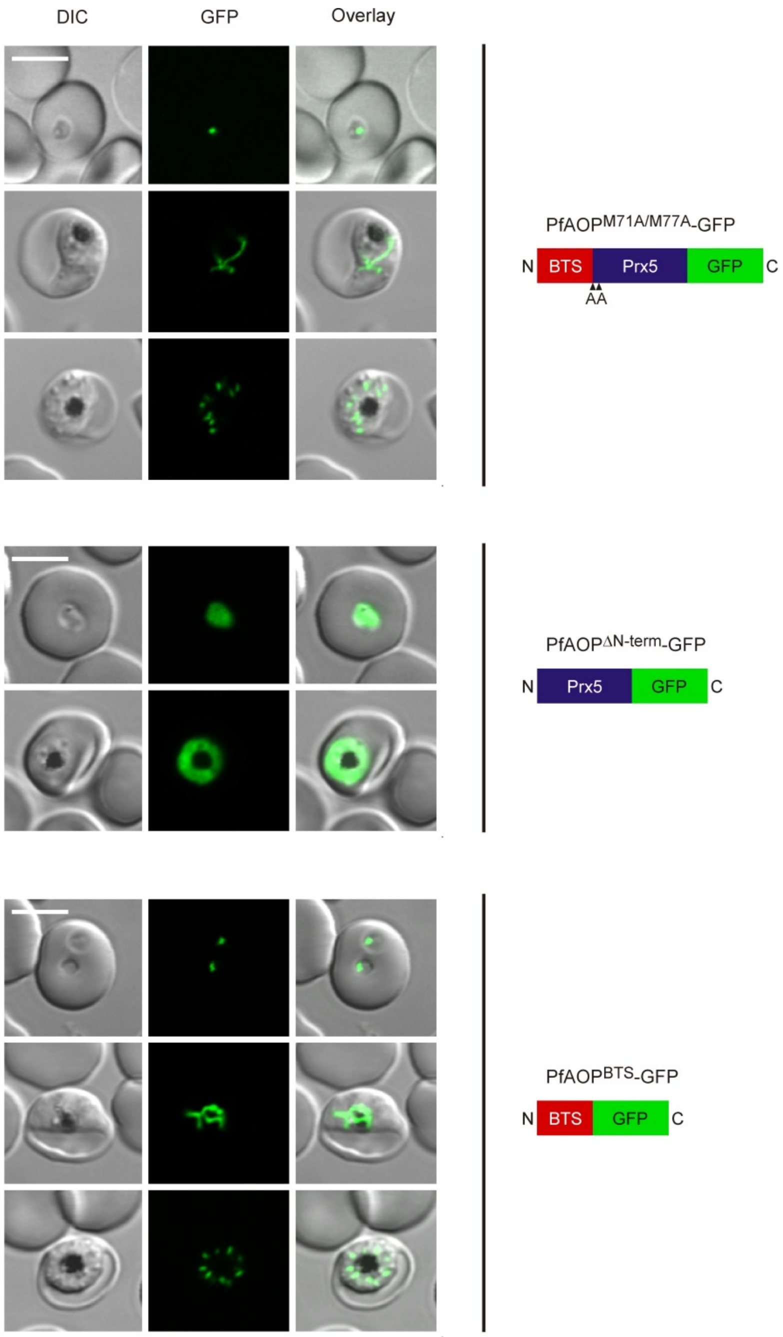

FIGURE S3: Cytosolic or apicoplast localization of mutated and truncated GFP-tagged PfAOP. Additional confocal live cell images of blood stage parasites expressing the indicated PfAOP-GFP chimera. Early trophozoite to late schizont stage parasites are shown from top to bottom for each construct. Scale bar, 5 µm.

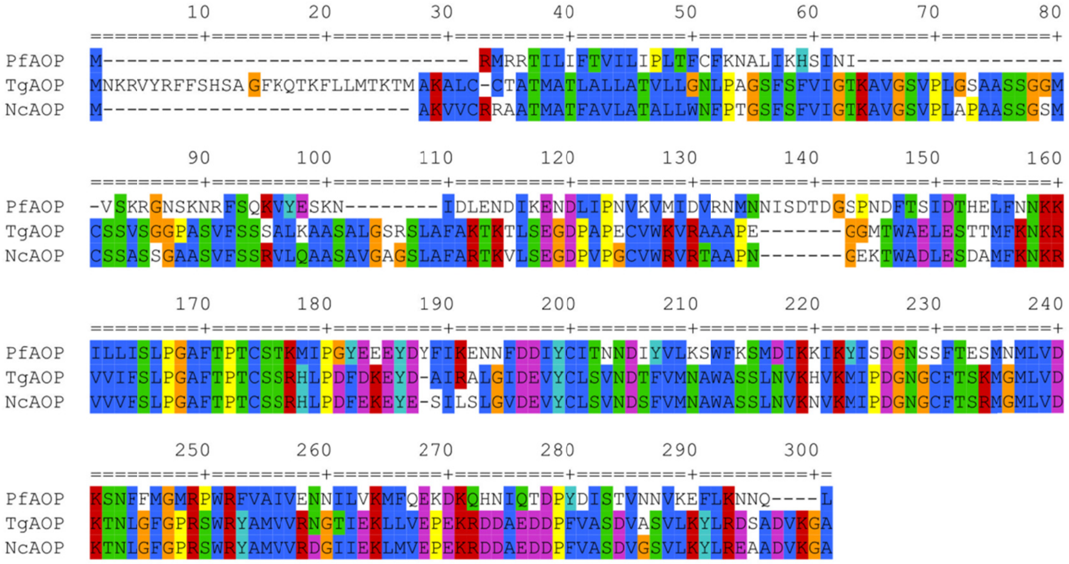

FIGURE S4: Apicomplexan Prx5 homologues have significantly altered N-termini. Multiple sequence alignment of the Prx5 homologues from *P. falciparum* PF3D7_0729200, *Toxoplasma gondii* TGGT1_038055 and *Neospora caninum* NCLIV_014020.
